# Acitretin Promotes the Differentiation of Myeloid-Derived Suppressor Cells in the Treatment of Psoriasis

**DOI:** 10.3389/fmed.2021.625130

**Published:** 2021-03-23

**Authors:** Panpan Liu, Cong Peng, Xiang Chen, Lisha Wu, Mingzhu Yin, Jie Li, Qunshi Qin, Yehong Kuang, Wu Zhu

**Affiliations:** ^1^The Department of Dermatology, Xiangya Hospital, Central South University, Changsha, China; ^2^Hunan Key Laboratory of Skin Cancer and Psoriasis, Changsha, China; ^3^Hunan Engineering Research Center of Skin Health and Disease, Changsha, China; ^4^Gerontology Center of Xiangya Hospital, Central South University, Changsha, China

**Keywords:** psoriasis, acitretin, MDSCs, M-MDSCs, differentiation, glutathione

## Abstract

Increased numbers of myeloid-derived suppressor cells (MDSCs) are involved in the development of psoriasis. Acitretin is used to treat psoriasis by regulating the proliferation and differentiation of keratinocytes, but little is known about the effect of acitretin on immune cells. Here, we reported that psoriasis patients had an expansion of MDSCs and monocytic-MDSCs (M-MDSCs) in peripheral blood and skin lesions. The number of MDSCs and M-MDSCs in peripheral blood correlated positively with disease severity. Acitretin could reduce the number of MDSCs and M-MDSCs in the peripheral blood of psoriasis patients as well as the spleen and skin lesions of IMQ-induced psoriasis-like model mice. Moreover, acitretin promoted the differentiation of MDSCs into macrophages, especially CD206^+^ M2 macrophages, and CD11c^+^MHC-II^+^ dendritic cells. Mechanically, acitretin dramatically increased the glutathione synthase (GSS) expression and glutathione (GSH) accumulation in MDSCs. Interruption of GSH synthesis abrogated the acitretin effect on MDSCs differentiation. Acitretin regulated GSS expression via activation of extracellular signal-regulated kinase 1/2. Thus, our data demonstrated a novel mechanism underlying the effects of acitretin on psoriasis by promoting MDSCs differentiation.

## Introduction

Psoriasis is an immune-mediated chronic inflammatory disease, affecting 2–3% of the population (1). The high proliferation and low differentiation of keratinocytes and dermal immune cells infiltration are the two major pathological manifestations of psoriasis (2, 3). The inflammatory effect induced by the interaction between keratinocytes and activated immune cells is also the main factor leading to the pathogenesis of psoriasis (4, 5). Acitretin, a synthetic retinoid belonging to the family of retinoid analogs (RA) drugs (6), has been used as the first-line treatment of psoriasis (7). It has been reported that acitretin could suppress the proliferation of keratinocytes and regulate their differentiation in the treatment of psoriasis (8), but it has little effect on Th1, Th17, and Tregs (9, 10). However, retinoic acid caused a pronounced inhibition of neutrophils in the treatment of pustular psoriasis (11), suggesting the anti-inflammatory effect of retinoids. Therefore, we sought to determine whether acitretin could regulate immunity in the treatment of psoriasis.

Myeloid-derived suppressor cells (MDSCs) are a heterogeneous population of immature cells, including immature granulocytes, monocytes, and dendritic cells (12). Human MDSCs are HLA-DR^−^CD11b^+^CD33^+^ and can be divided into two major subsets, CD15^−^CD14^+^ monocytic MDSCs (M-MDSCs), and CD15^+^CD14^−^ granulocytic MDSCs (G-MDSCs) (13, 14). Murine MDSCs are Gr-1^+^CD11b^+^ and can be further subdivided into CD11b^+^Ly6G^−^Ly6C^+^ M-MDSCs and CD11b^+^Ly6G^+^Ly6C^−^ G-MDSCs (15). Traditionally, MDSCs have been studied in regard to their increased numbers in cancer patients and immunosuppressive functions (12, 16). Recent research focused on the pathologic role of expanding MDSCs in inflammatory diseases and autoimmune diseases, such as multiple sclerosis, rheumatoid arthritis, inflammatory bowel disease, autoimmune hepatitis, and psoriasis (17–19). In addition, the increased numbers of MDSCs in inflammatory diseases presented a pro-inflammatory role and impaired immunosuppressive function (20). The MDSCs from patients with active systemic lupus erythematosus or rheumatoid arthritis showed the induction of the Th17 response and Th17 differentiation (21, 22). Psoriatic MDSCs could produce increased IL-23, IL-1β, and CCL4 cytokines, were unable to suppress T-cell proliferation, displayed decreased expression levels of PD-1 as well as PD-L1, and failed to produce Tregs (23–26). All-trans retinoic acid (ATRA), a member of the retinoid family, potently eliminated MDSCs in cancer patients (27). Therefore, we investigated whether acitretin could regulate MDSCs.

In this study, we found psoriasis patients have a significant increase in MDSCs and M-MDSCs populations that correlated positively with disease severity. Acitretin reduced the number of MDSCs and M-MDSCs in the peripheral blood of psoriasis patients and spleen and skin lesions of imiquimod (IMQ)-induced model mice of psoriasis. Furthermore, we found that acitretin promoted the differentiation of MDSCs via increasing glutathione accumulation, which were activated by the ERK1/2 MAPK signaling pathway. In summary, these findings indicated that acitretin promoted the differentiation of MDSCs in the treatment of psoriasis.

## Materials and Methods

### Human Subjects

All patients in this study were diagnosed with plaque psoriasis by a dermatologist based upon clinical presentation or histologic examination. Patients who were treated with any treatment in the past 3 months were excluded from this study. Psoriasis disease activity was assessed using the psoriasis area and severity index (PASI) score (28). Healthy volunteers were randomly recruited with matched age and gender of psoriasis patients. Peripheral blood mononuclear cells (PBMCs) were collected from 77 patients with plaque psoriasis and 30 healthy controls. The skin was collected from 20 patients with plaque psoriasis and 9 healthy controls. Seventeen psoriasis patients were treated with acitretin (HUAPONT PHARM, Chongqing, China) 30 mg/d for 8 weeks with the PASI score significantly improved, and PBMCs were collected before and after the treatment. Patient information was shown in Supplementary Tables 1, 2, 3. For all the experiments using clinical samples, we have ensured the blinded outcome assessment. All human studies were approved by the ethics committees of Xiangya hospital of Central South University, Changsha, Hunan, China, and informed consent was obtained from all subjects.

### IMQ-Induced Psoriasis-Like Model Mice

8-week-old BALB/c female mice (purchased from the department of laboratory animals of Central South University) were used. A daily dose of 62.5 mg of 5% imiquimod (IMQ) cream (Med-shine Pharmaceutical Co., Ltd., Sichuan, China) was applied to the shaved back of mice for 6 consecutive days (29). Mice were treated with acitretin (5 mg/kg, daily) (HUAPONT PHARM, Chongqing, China) by oral administration once per day. All animal experiments were performed according to the Animal Care and Use Committee guidelines of Xiangya medicine school of Central South University.

### Histological Evaluation

Human and mouse skin tissues were embedded in paraffin and split for routine histopathology on paraffin slicing machine-cut 3 mm sections. Sections were stained with hematoxylin and eosin (H&E stain) for histological evaluation.

### Measurement of Skin Scores and Epidermal Thickness

The clinical skin scores of mice were determined from day 1 (the 1st day of IMQ treatment) and every other day until day 7 using the modified PASI as previously described (30, 31). The degree of skin erythema, induration, and scale was classified as follows: 0, no symptoms; 1, mild; 2, moderate; 3, severe; or 4, very severe. The thickness of the epidermis was measured from the stratum basale to the stratum granulosum using Image Pro-Plus (Image Pro-Plus 6.0 image-analysis software). The average value from seven random fields of view was calculated for each mouse.

### Immunohistochemistry and Immunohistochemical Analysis

Immunohistochemistry was performed according to a previous study (32). Briefly, sections were incubated with monoclonal antibody: PCNA (Abcam, Cat. ab15497), K17 (Abcam, Cat. ab109725), K10 (Abcam, Cat. ab76318), CD86 (NOVUS, Cat. NBP2-25208), CD206 (Abcam, Cat. ab64693), or MHC-II (Abcam, Cat. ab55152) at 4°C overnight. Bound antibodies were detected by using a conventional streptavidin-biotin method according to the manufacturer's instructions (ZSGB-BIO Cat. PV-9000). The reaction was visualized by DAB+ Chromogen, and slides were counterstained with hematoxylin. For immunohistochemical analysis, immune-stained sections were characterized semi-quantitatively by digital image analysis using the Image Pro-Plus (Image Pro-Plus 6.0 image-analysis software) by using the method as previously reported (33, 34). Briefly, images at 1,360 × 1,024-pixel resolution at 400× magnification were obtained with an Olympus CX41 microscope fitted with a micro image video camera (Mshot). A series of seven random images on several sections were taken for each immune-stained parameter to obtain a mean value for statistical comparison. Staining was defined via color intensity, and a color mask was made. The mask was then applied equally to all images, and measurements were obtained. The measurement parameter included integrated optical density (IOD) and the area. The optical density was calibrated, and the area of interest was set through: PCNA (hue 9–36, saturation 0–255, intensity 0–241), K17 (hue 10–31, saturation 0–255, intensity 0–170), K10 (hue 15–31, saturation 0–255, intensity 0–170), CD86 (hue 9–70, saturation 0–255, intensity 0–180), CD206 (hue 9–70, saturation 0–255, intensity 0–196), MHC-II (hue 9–115, saturation 0–255, intensity 0–190), and then the values were counted. Two independent examiners evaluated these sections without prior knowledge of the clinical status. PCNA IOD, K17 IOD, K10 IOD/ Area, CD86 IOD, CD206 IOD, or MHC-II IOD was calculated.

### Cell Isolation

PBMCs were prepared by density gradient centrifugation using Lymphocyte Separation Medium (human). Single-cell suspensions of the mice were prepared from the spleen, and red blood cells were removed using Lysing Buffer (BD, Cat. 555899). Skin lesions were dissected and digested with 2.0 mg/mL collagenase IV (Sigma-Aldrich, Cat. V900893) and 1.0 mg/mL dispase II (Sigma-Aldrich, Cat. D4693) for 60 min at 37°C. All single-cell suspensions are filtered through 40-micron pores (BD, Cat. 352340). Gr-1^+^ MDSCs were isolated by using biotinylated anti-Gr-1 antibody (Miltenyi Biotec, Cat.130-101-849) and streptavidin microbeads (Miltenyi Biotec, Cat.130-048-102) with MiniMACS columns, and the purity of the cells after separation was >95%.

### Flow Cytometric Analysis

Flow cytometry was used to determine the phenotypes of human and mouse MDSCs, macrophages, and dendritic cells. Cells were incubated with live/dead stain (Zombie Aqua™ Fixable Viability Kit; BioLgend Cat. 432102) and Fc block (BioLegend Cat. 101302). Cells were then washed and stained for using various combinations of the following fluorochrome-conjugated mAbs: anti-human HLA-DR (L243), CD11b (ICRF44), CD33 (P67.6), CD15 (HI98), CD14 (63D3), and anti-mouse Gr-1 (RB6-8C5), CD11b (M1/70), Ly6G (1A8), Ly6C (HK1.4), F4/80 (BM8), CD86 (BU63), MHC-II (39-10-8), and CD11c (N418) from Biolegend (San Diego, USA). For intracellular staining, cells were fixed and permeabilized using the Foxp3/Transcription Factor Staining Buffer Kit (eBioscience Cat. 00-5523-00) according to the manufacturer's protocol. Cells were stained intracellularly with anti-CD206 (C068C2) antibody. All samples were detected on FACSCalibur (BD, California, USA) and analyzed by FlowJo software (version 10.0.7). Isotype-matched antibodies were used with all the samples as controls.

### Differentiation of MDSCs

MDSCs were isolated from the bone marrow of IMQ-induced model mice, resuspended in RPMI 1640 (Biological Industries Cat. 01-100-1ACS) supplemented with 10% FBS (Gibco Cat. 16140071), HEPES (Gibco Cat. 15630080), sodium pyruvate (Gibco Cat. 11360-070), Non-Essential Amino Acids Solution (Gibco Cat. 11140050), 2-Mercaptoethanol (Gibco Cat. 21985023) and 20 ng/mL murine GM-CSF (PeproTech, Cat. 96-315-03-20), and plated at concentration 1.0 × 10^6^/mL in 24-well plate. MDSCs were cultured for 4–5 days. Acitretin (HUAPONT PHARM, Chongqing, China), sulfasalazine (SAS) (MCE, Cat. HY-14655), or selumetinib (MCE, Cat. HY-50706) was added on days 1 and 3. After 4–5 days of culture, cells were collected, and the presence of different cell populations was evaluated by flow cytometry.

### RNA Seq Analysis

Total RNA was isolated and reverse-transcribed into cDNA to generate an indexed Illumina library, followed by sequencing at the Shenzhen Genomics Institute (Shenzhen, China) using a BGISEQ-500 platform. High-quality reads were aligned to the mouse reference genome (GRCm38) by Bowtie2. The expression of individual genes was normalized to fragments per kilobase of the exon model per million mapped reads from RNA-Seq by Expectation Maximization. Significant differential expression was set if a gene with > 2-fold expression difference vs. the control with an adjusted *p*-value of < 0.05. The differentially expressed genes (DEGs) were analyzed by gene ontology using AMIGO and DAVID software. The enrichment degrees of DEGs were analyzed using the Kyoto Encyclopedia of Genes and Genomes (KEGG) annotations.

### RT-qPCR

RNA was extracted from cells using TRIpure Reagent (Bioteke Cat. RP1001), according to the manufacturer's instructions. RNA was converted to cDNA using HiScript II Q RT SuperMix for qPCR (+gDNA wiper) (Vazyme Cat. R223-01), and gene expression was determined by RT-qPCR using the UltraSYBR One-Step RT-qPCR Kit (CWBIO Cat. CW0659) on a 7,500 Fast thermocycler (Applied Biosystems). The relative expression of target genes was confirmed using the quantity of target gene/quantity of β-Actin. The fold change of gene expression was calculated by 2^−(ΔCt experimental group−ΔCt control group)^, which normalized to the control group. The primer sequences used for RT-qPCR were as follows: *Gss*: forward, 5′ -CTGATGCTAGAGAGATCTCGTG-3′, and reverse, 5′ -TTCACCCATGTCCAGTGAATAG-3′; β*-Actin*: forward, 5′ -GCTCTGGCTCCTAGCACCAT-3′, and reverse, 5′ -GCCACCGATCCACACAGAGT-3′. All primers were purchased from Sangon Biotech.

### Western Blotting

Cells were lysed in radio immunoprecipitation assay buffer supplemented with protease and phosphatase inhibitor (Bimake Cat. B14002). The protein concentration had been tested with a BCA Kit (Bimake Cat. PP1002), and appropriate amounts of protein were prepared for SDS-PAGE and then transferred to a PVDF membrane (Millipore). The membranes were blocked for 1 h with 5% bovine serum albumin (BSA) at room temperature and then incubated with primary antibodies overnight at 4°C. The membranes were washed with phosphate-buffered saline (PBS) buffer with 0.1% Tween 20 (PBS-T), reacted with horseradish peroxidase-conjugated secondary antibodies for 1 h, and visualized using an enhanced chemiluminescence substrate. Membranes were visualized using WesternBright ECL HRP substrate (Advansta) on a GelDoc system (Bio-Rad). Images were analyzed with the Image Lab software (Bio-Rad). Rabbit anti-GSS Ab (1:500; ABclonal Cat. ab11557), rabbit anti-p-MEK1/2 (Ser217/221) Ab (1:1000; CST Cat. 9154), rabbit anti-MEK1/2 Ab (1:1000; CST Cat. 9126), rabbit anti-p44/42 MAPK (ERK1/2) (Thr202/Tyr204) Ab (1:1000; CST Cat. 4370), Rabbit anti-p-p38 MAPK (Tyr182) Ab (1:1000; Santa Cruz Biotechnology Cat. sc-166182), or mouse anti-GAPDH Ab (1:2000, Proteintech Cat. 6004-1-Ig) was used.

### Measurement of GSH and Reactive Oxygen Species (ROS)

MDSCs were isolated from the bone marrow of IMQ-induced model mice, resuspended in 20 ng/mL murine GM-CSF (PeproTech, Cat. 96-315-03-20), and plated at concentration 1.0 × 10^6^/mL in 24-well plate. MDSCs were treated with acitretin 500 ng/mL, SAS 200 μM or vehicle control for 48 h and then collected for the measurement of GSH or ROS. GSH level was determined using GSH Assay Kit (Beyotime Cat. S0053) according to the manufacturer's protocol. Absorbance was read at 412 nm using a microplate reader. GSH level was expressed as nanograms per 10^6^ cells. For the measurement of ROS, cells were collected and then loaded with DCFH-DA (Solarbio Cat. CA1410) in RPMI 1640 at 37°C and incubated for 20 min according to the manufacturer's instructions. Excess DCFH-DA was removed by washing with RPMI 1640. The ROS levels were measured by flow cytometry and analyzed using the FlowJo software.

### Statistical Analysis

Statistical analyses were performed on GraphPad Prism 8.0 software. Data are expressed as means ± SEM. A Student's *t* test was used to compare two conditions, and an analysis of variance (ANOVA) with Bonferroni or Newman-Keuls correction was used for multiple comparisons. Correlation analysis was performed with Pearson Correlation Test. The level of significance was defined as *p* < 0.05. ^*^p < 0.05; ^**^p < 0.01; ^***^p < 0.001; ^****^p < 0.0001.

## Results

### MDSCs and M-MDSCs Expansion Was Found in the Peripheral Blood and Skin Lesions of Psoriasis Patients

**Figure 1:**
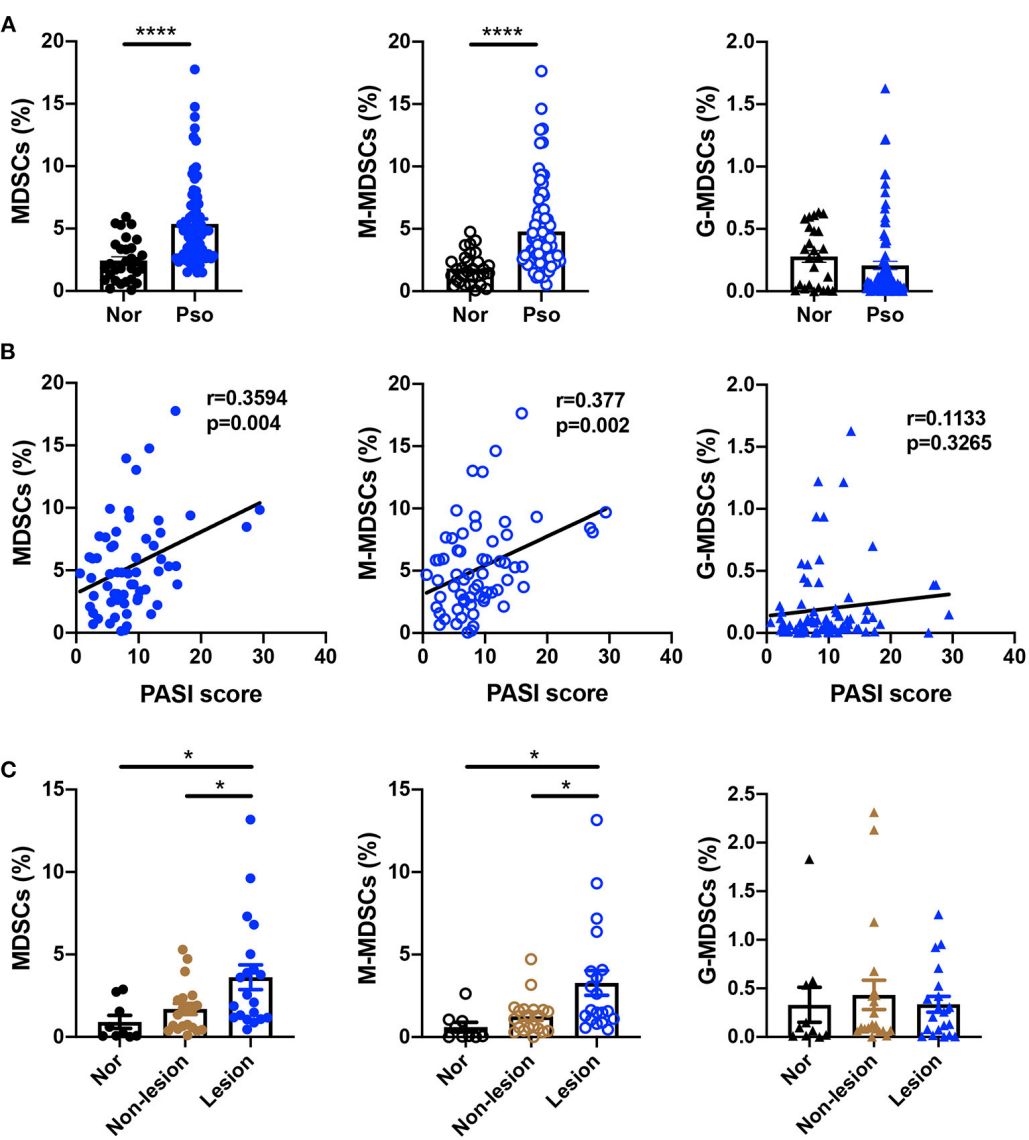
MDSCs and M-MDSCs expansion was found in the peripheral blood and skin lesions of psoriasis patients. **(A,B) (A)** Percentages of HLA-DR^−^CD11b^+^CD33^+^ MDSCs (left panel), HLA-DR^−^CD11b^+^CD33^+^CD15^−^CD14^+^ M-MDSCs (middle panel), and HLA-DR^−^CD11b^+^CD33^+^CD15^+^CD14^−^ G-MDSCs (right panel) in PBMCs of healthy controls (Nor) (*n* = 30) and plaque psoriasis (Pso) (*n* = 77), and **(B)** the correlation analysis between the indicated cells frequency and disease activity (that is, PASI score) in plaque psoriasis. **(C)** The number of MDSCs (left panel), M-MDSCs (middle panel), and G-MDSCs (right panel) in normal skins (*n* = 9), non-lesion tissues (*n* = 20), and skin lesion of psoriasis (*n* = 20). Data represent the mean ± SEM. *p < 0.05, ****p < 0.0001.

To confirm the number of MDSCs in the psoriasis patients, we first measured the percentage of MDSCs and their subsets in PBMCs isolated from healthy controls and psoriasis patients. The characteristics of psoriasis patients and healthy subjects were shown in Supplementary Table 1. MDSCs were defined as HLA-DR^−^CD11b^+^CD33^+^, which were further divided into CD15^−^CD14^+^ M-MDSC and CD15^+^CD14^−^ G-MDSC subsets (Supplementary Figure 1). Compared to the healthy control subjects, the plaque psoriasis patients showed significant increases in the percentages of both MDSCs and M-MDSCs (Figure 1A), which were positively correlated with disease severity assessed by PASI score (Figure 1B). However, there was no significant difference in the percentage of G-MDSCs between the groups (Figures 1A,B). In addition, the number of MDSCs and M-MDSCs in the skin lesions of the psoriasis patients was markedly higher than that in the non-lesion tissue and normal skin (Figure 1C, Supplementary Table 2). There was no significant difference in the percentage of G-MDSCs between these groups in skin lesions (Figure 1C). Therefore, the number of MDSCs, especially M-MDSCs, in peripheral blood and skin lesion of psoriasis patients was significantly higher than that of healthy controls.

### Acitretin Deceased the Number of MDSCs and M-MDSC *in vivo*

**Figure 2:**
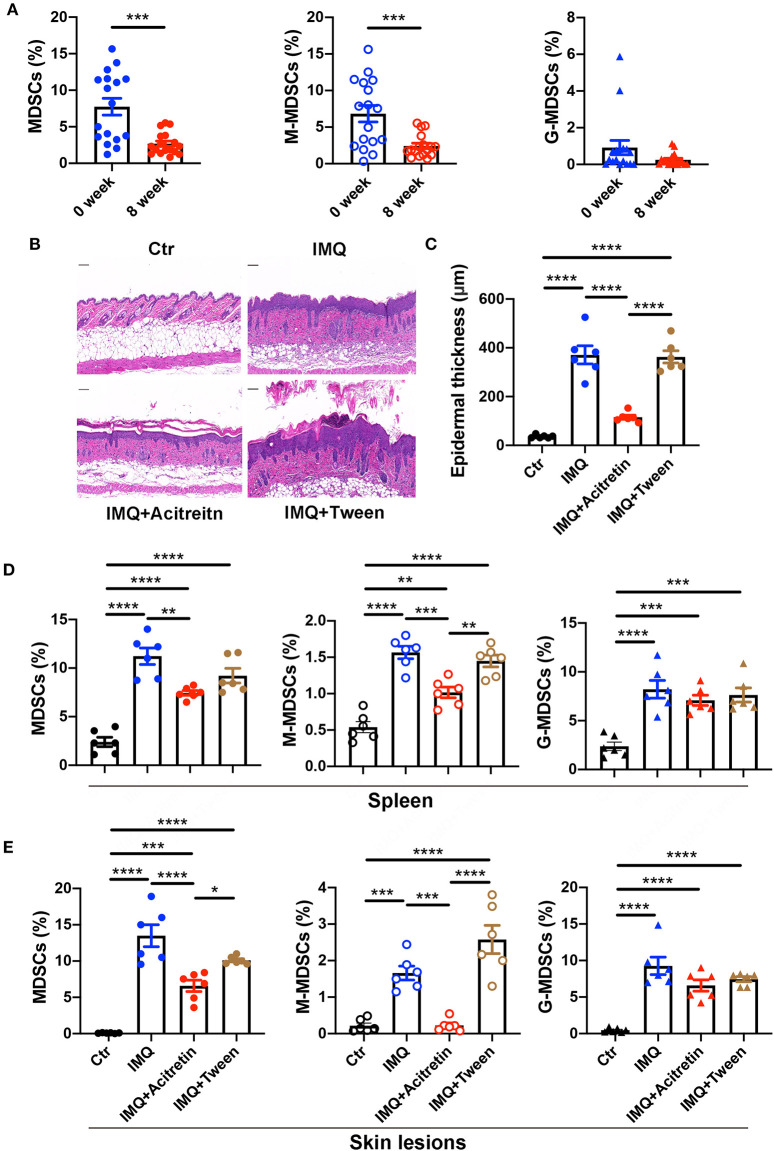
Acitretin deceased the number of MDSCs and M-MDSC *in vivo*. **(A)** The percentages of MDSCs (left panel), M-MDSCs (middle panel), and G-MDSCs (right panel) in PBMCs of psoriasis patients before and after the treatment of acitretin for 8 weeks (*n* = 17). IMQ-induced psoriasis-like model mice treated with oral acitretin or tween (solvent) once per day for 6 days. **(B)** The H&E staining of the back skin derived from Control (Ctr) and IMQ-induced model mice treated with acitretin or tween (solvent) (*n* = 6). Scale bars: 100 μm. **(C)** The epidermal thickness of mice in **B** (*n* = 6). **(D,E)** The statistical data of Gr-1^+^CD11b^+^ MDSCs (left panel), CD11b^+^Ly6G^−^Ly6C^+^ M-MDSCs (middle panel), and CD11b^+^Ly6G^+^Ly6C^−^ G-MDSCs (right panel) in the spleen **(D)** and skin lesions **(E)** of IMQ-induced psoriasis-like model mice treated with oral acitretin or tween (solvent) (*n* = 6). All results represent at least 3 independent experiments. Data represent the mean ± SEM. *p < 0.05, **p < 0.01, ***p < 0.001, ****p < 0.0001.

We then tried to determine whether acitretin reduced the number of MDSCs in the treatment of psoriasis. We measured the percentage of MDSCs, M-MDSCs, and G-MDSCs in the PBMCs of psoriasis patients treated with acitretin for 8 weeks with the PASI score significantly improved. The characteristics of the psoriasis patients who were treated with acitretin were shown in Supplementary Table 3. The number of MDSCs and M-MDSCs in the peripheral blood of the psoriasis patients was significantly decreased after acitretin treatment (Figure 2A). There was no significant difference in the percentage of G-MDSCs after acitretin treatment (Figure 2A).

To determine whether acitretin has the same effect on MDSCs in the IMQ-induced model mice of psoriasis, we first treated IMQ-induced psoriasis-like model mice with oral acitretin once per day. After the IMQ-induced model mice were treated with acitretin for 6 days, the scaling and thickness of the skin on the back of the mice were significantly alleviated, which was confirmed by the histological evaluation showing a significant decrease in epidermal thickness; the PASI score was also significantly decreased (Figures 2B,C, Supplementary Figures 2A–D). Besides, the expression of PCNA and K17 (the makers of cell proliferation) significantly decreased in the skin lesion of the acitretin treatment group. In contrast, the expression of K10 (the markers of keratinization) increased in the skin lesion of the acitretin treatment group compared with the IMQ groups (Supplementary Figures 2E–G). We then measured the percentage of MDSCs, M-MDSCs, and G-MDSCs in the spleen and skin lesions of acitretin-treated IMQ-induced psoriasis-like model mice. The results showed that the number of Gr-1^+^CD11b^+^ MDSCs, CD11b^+^Ly6G^−^Ly6C^+^ M-MDSCs, and CD11b^+^Ly6G^+^Ly6C^−^ G-MDSCs significantly increased in the spleen and skin lesions of IMQ-induced model mice compared with control group mice (Figures 2D,E, Supplementary Figure 3). The number of MDSCs and M-MDSCs in the spleen and skin lesions was decreased significantly in the acitretin treatment group compared with the IMQ groups (Figures 2D,E). However, there was no significant difference in the number of G-MDSCs after acitretin treatment (Figures 2D,E). Therefore, these results indicated that acitretin reduced the number of MDSCs and M-MDSCs in the psoriasis patients and psoriasis-like model mice.

### Acitretin Promoted the Differentiation of MDSCs

**Figure 3:**
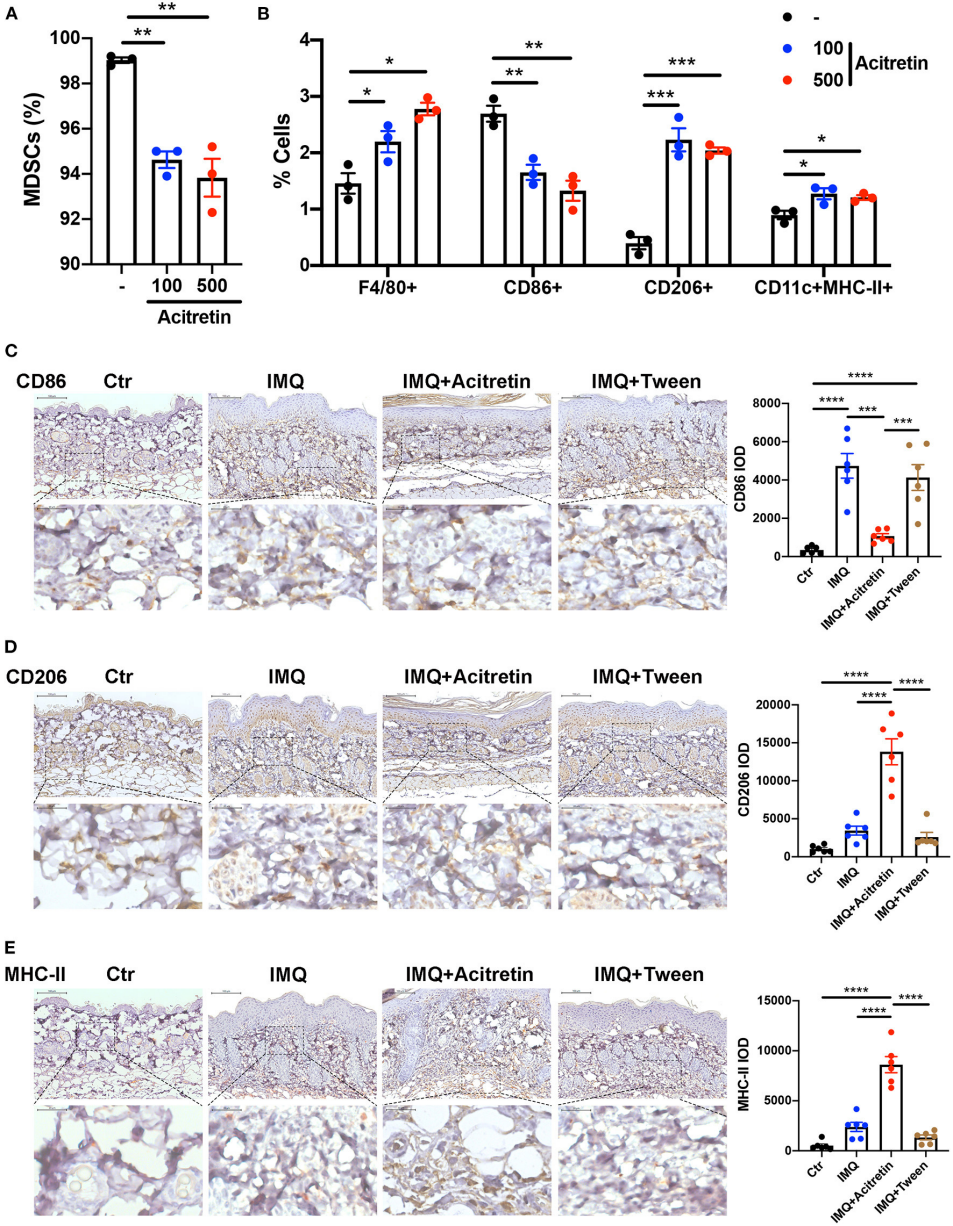
Acitretin promoted the differentiation of MDSCs. **(A,B)** MDSCs were isolated from the bone marrow of IMQ-induced model mice and cultured for 4 days with 20 ng/mL GM-CSF. Acitretin (100 ng/mL or 500 ng/mL) or vehicle control (-) was added on days 1 and 3. **(A)** The percentages of MDSCs treated with acitretin. **(B)** The effect of acitretin on differentiation of MDSCs into F4/80^+^ macrophage, CD86^+^ M1 macrophage, CD206^+^ M2 macrophage, and CD11c^+^MHC II^+^ dendritic cells. The presence of different cell populations was evaluated by flow cytometry. **(C–E)** IMQ-induced psoriasis-like model mice treated with oral acitretin or tween (solvent) once per day for 6 days. Paraffin sections of the back skin of Control (Ctr), IMQ, IMQ+Acitretin, and IMQ+Tween group were stained for CD86, CD206, and MHC-II by immunohistochemistry. CD86 IOD, CD206 IOD, and MHC-II IOD measured by image pro plus 6.0 expressed the CD86, CD206, and MHC-II expression. **(C)** CD86 stain, **(D)** CD206 stain, **(E)** MHC-II stain. Scale bars: 100 μm (upper panel), scale bars: 20 μm (lower panel). Statistical data are shown in the right panel. All results represent at least three independent experiments. Data represent the mean ± SEM. *p < 0.05, **p < 0.01, ***p < 0.001, ****p < 0.0001.

MDSCs are immature cells and have the ability to differentiate into macrophages and dendritic cells (DCs) (35). To test whether acitretin affected the differentiation of MDSCs, Gr-1^+^ MDSCs were isolated from the bone marrow of IMQ-induced model mice and cultured for 4 days with GM-CSF. Acitretin (100 ng/mL or 500 ng/mL, considering that the concentration of acitretin in human blood is 196–728 ng/mL) was added on days 1 and 3. The results showed that acitretin substantially reduced the percentage of MDSCs (Figure 3A) and increased the proportion of F4/80^+^ macrophages, especially CD206^+^ M2 macrophages (Figure 3B, Supplementary Figure 4). However, the percentage of CD86^+^ M1 macrophages was slightly decreased after acitretin treatment (Figure 3B, Supplementary Figure 4). In addition, acitretin increased the proportion of CD11c^+^MHC-II^+^ dendritic cells (Figure 3B, Supplementary Figure 4). To clarify the effect of acitretin on the differentiation of MDSCs *in vivo*, we analyzed the expression of macrophages and dendritic cells in the skin lesion of IMQ-induced model mice treated with acitretin by immunohistochemistry. The results showed that the expression of CD86 significantly decreased in the skin lesion of the acitretin treatment group, while the expression of CD206 and MHC-II increased in the skin lesion of the acitretin treatment group compared with the IMQ groups (Figures 3C–E). Thus, these data indicated that acitretin induced the differentiation of MDSCs into macrophages, especially CD206^+^ M2 macrophages, and CD11c^+^MHC-II^+^ dendritic cells.

### Mechanism of Acitretin Effect on the Differentiation of MDSCs

**Figure 4:**
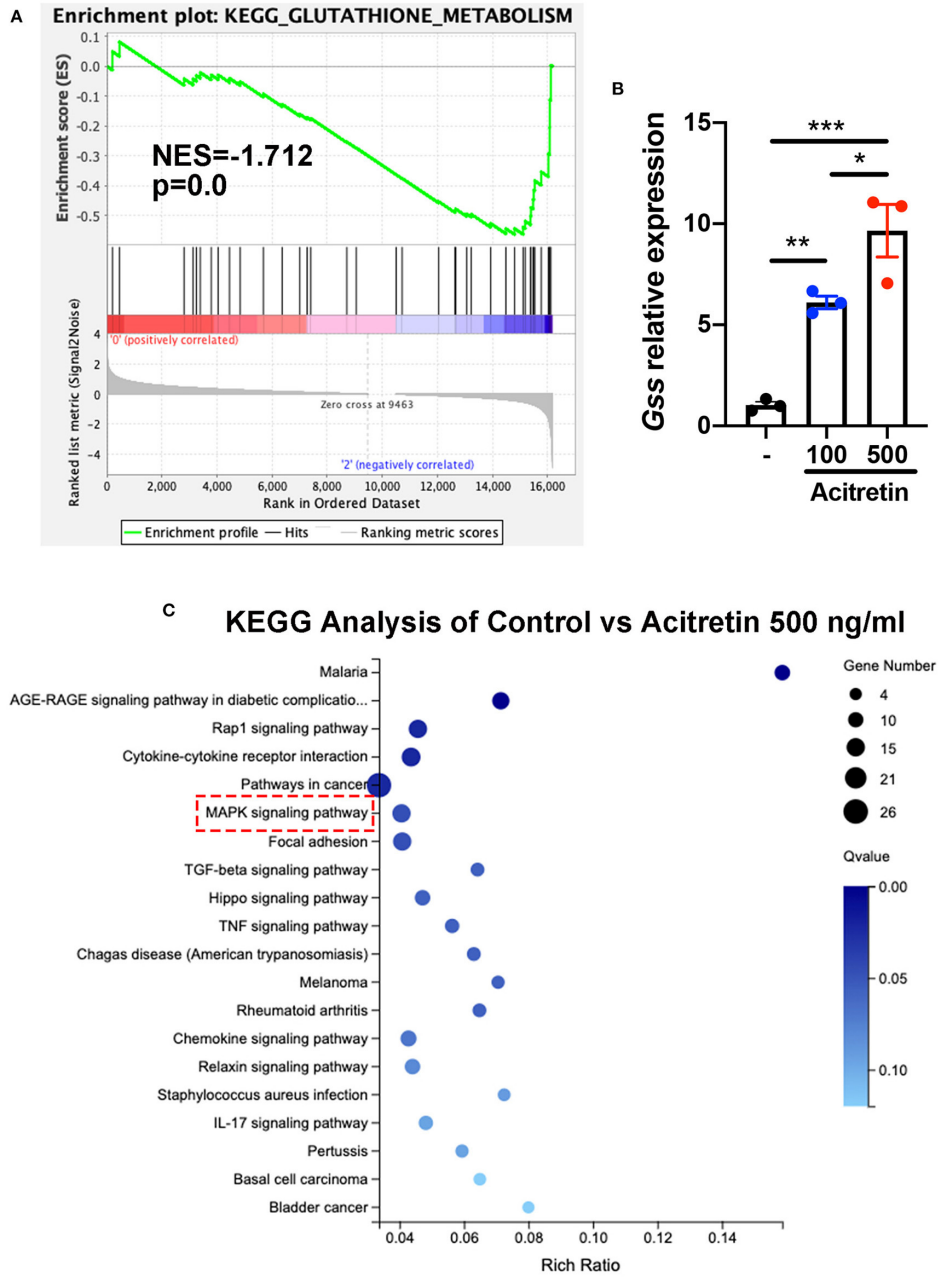
Transcriptome analysis of MDSCs treated with acitretin. MDSCs were isolated from the bone marrow of IMQ-induced model mice and cultured in the presence of 20 ng/mL GM-CSF with or without acitretin 500 ng/mL. After 24 h of treatment, MDSCs were collected and used for whole-genome transcriptome analysis. **(A)** Glutathione metabolism enriched by GSEA identified from RNA-seq data of control vs. acitretin group. Normalized enrichment score (NES) and Normalized *p*-value (p) are shown in the plot. **(B)** MDSCs were treated with control (-), acitretin 100 ng/mL, or acitretin 500 ng/mL for 48 h. Expression levels of *Gss* were examined by RT-qPCR. The results are representative of at least three independent experiments with three samples per group in each. Data represent the mean ± SEM. *p < 0.05, **p < 0.01, ***p < 0.001. **(C)** KEGG pathways analysis of differentially expressed genes in MDSCs treated with control or acitretin 500 ng/mL for 24 h.

To investigate the mechanisms of acitretin effect on the differentiation of MDSCs, MDSCs isolated from IMQ-induced psoriasis-like model mice treated with control or acitretin 500 ng/mL for 24 h was performed using RNA-seq. Using gene set enrichment analysis (GSEA) to identify the Kyoto Encyclopedia of Genes and Genomes (KEGG) pathways, we found the top three enriched pathways included glutathione metabolism (Figure 4A). And RT-qPCR analysis confirmed that the expression of glutathione synthase (GSS) in MDSCs was significantly increased by treatment with acitretin (Figure 4B). Furthermore, among 16,183 changed genes between the control group and acitretin group, 317 were differentially expressed genes (DEGs; |log_2_FC|> 1.0 and *p* < 0.001). The KEGG pathways enrichment analysis highlighted that the MAPK signaling pathway was activated after the treatment of acitretin in MDSCs (Figure 4C), an essential signaling cascade that controls cell proliferation, survival, and differentiation (36).

**Figure 5:**
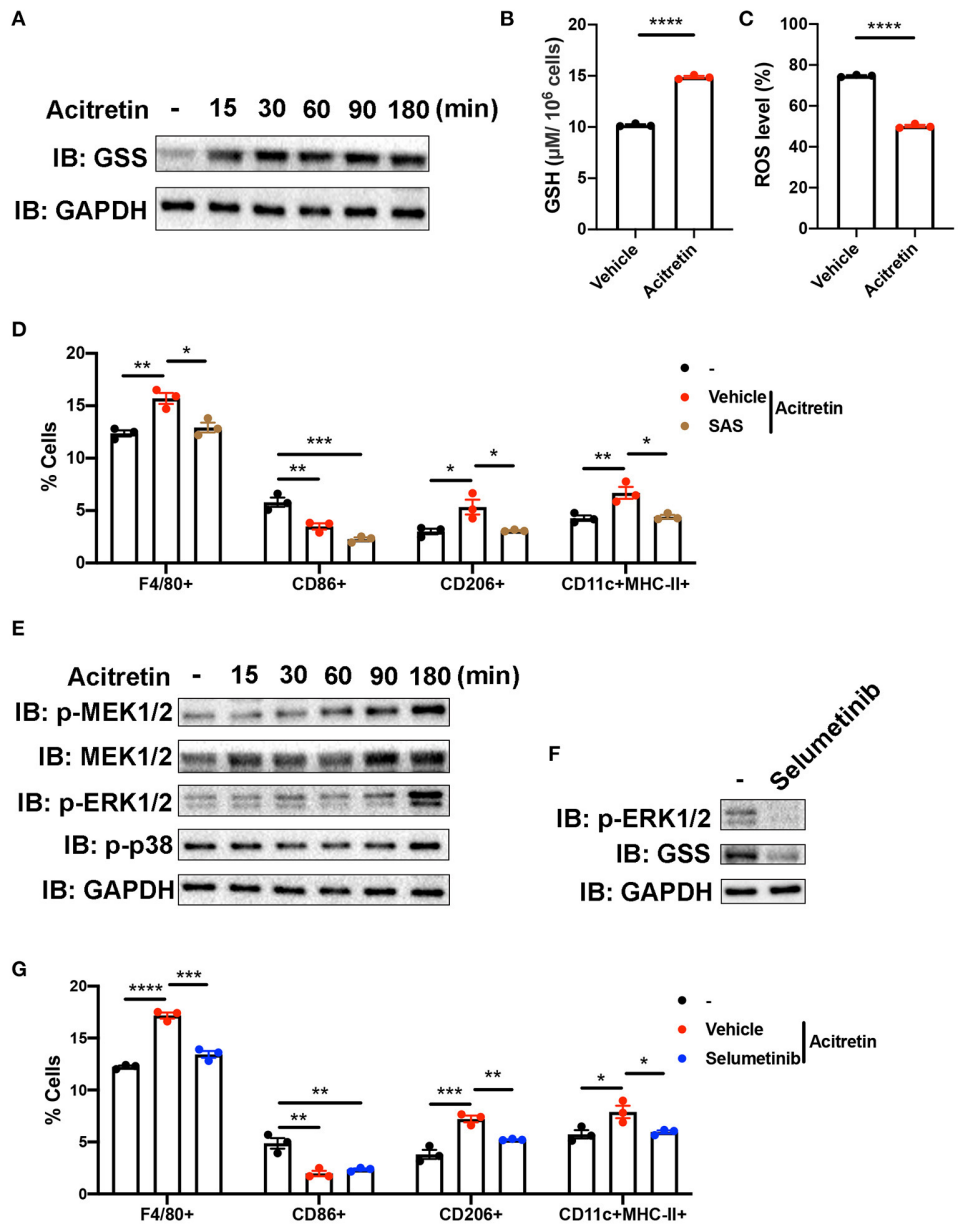
Mechanism of acitretin effect on the differentiation of MDSCs. **(A)** The effect of acitretin on GSS. Gr-1^+^MDSCs were isolated from the bone marrow of IMQ-induced model mice and cultured in the presence of 20 ng/mL GM-CSF with or without 500 ng/mL acitretin for 15, 30, 60, 90, or 180 min. Whole-cell lysates were obtained, and the protein expression of GSS was evaluated in Western blotting as described in **Materials and Methods**. GAPDH was blotted as a loading control. **(B,C)** MDSCs from IMQ-induced model mice were treated with vehicle control or 500 ng/mL acitretin for 48 h as described above. MDSCs were obtained, and the level of GSH was measured with a GSH detection kit. The level of ROS was detected by flow cytometry. **(B)** The level of GSH in MDSCs. **(C)** The level of ROS in MDSCs. **(D)** The effect of SAS combination with acitretin on the differentiation of MDSCs. MDSCs were isolated from the bone marrow of IMQ-induced model mice and cultured for 5 days with 20 ng/mL GM-CSF. Acitretin 500 ng/mL or SAS 200 μM was added on days 1 and 3. The presence of different cell populations was evaluated by flow cytometry. **(E)** Western blot analysis of different proteins in MDSCs after treatment with acitretin. **(F)** The effect of selumetinib on MDSCs. MDSC were isolated from IMQ-induced model mice and cultured in the presence of 20 ng/mL GM-CSF with or without 10 nM specific MEK1/2 inhibitor selumetinib for 3 h. Cell lysates were prepared, and the protein expression of GSS and p-ERK1/2 was evaluated in Western blotting. GAPDH was blotted as a loading control. **(G)** The effect of selumetinib combination with acitretin on the differentiation of MDSCs. MDSCs were isolated from the bone marrow of IMQ-induced model mice and cultured for 5 days with 20 ng/mL GM-CSF. Acitretin 500 ng/mL or selumetinib 10 nM was added on days 1 and 3. The presence of different cell populations was evaluated by flow cytometry. Data represent the mean ± SEM. *p < 0.05, **p < 0.01, ***p < 0.001, ****p < 0.0001.

GSS involved in the synthesis of glutathione (GSH), an important antioxidant in mammalian cells (37). Because the increased level of ROS contributed to the inability of MDSCs differentiation (38), we intended to explore whether acitretin promoted the differentiation of MDSCs by regulating glutathione metabolism. To address this hypothesis, we isolated MDSCs from the bone marrow of IMQ-induced model mice and cultured cells in the presence of GM-CSF with or without acitretin. We found the protein level of GSS up-regulated in MDSCs exposed to acitretin and was observed as early as 15 min after the start of the treatment with acitretin (Figure 5A). The up-regulated expression of GSS is related to the increased level of GSH, so we measure the GSH level in MDSCs by using an enzymatic assay. The results showed that acitretin increased the level of GSH in MDSCs (Figure 5B). Besides, we found the ROS level significantly decreased in MDSCs after the treatment of acitretin (Figure 5C, Supplementary Figure 5A), indicating that acitretin-induced the increased level of GSH neutralized the ROS production of MDSCs. Sulfasalazine (SAS) is an inhibitor of system ${\text{x}}_{\text{c}}^{-}$ cystine/glutamate antiporter, which is required for the GSH synthesis (39). 48 h treatment of MDSCs isolated from the bone marrow of IMQ-induced model mice with SAS dramatically decreased the level of GSH and resulted in the accumulation of ROS (Supplementary Figures 5B,C). To investigate whether SAS interfered with the effect of acitretin on MDSCs differentiation, MDSCs were isolated from the IMQ-induced model mice and cultured for 5 days with GM-CSF and acitretin with or without SAS. The results showed that in the presence of SAS, acitretin had no effect on the proportion of F4/80^+^ macrophages, CD206^+^ M2 macrophages, and CD11c^+^MHC II^+^ dendritic cells, although still decreased the percentage of CD86^+^ M1 macrophages (Figure 5D). Therefore, acitretin-induced the increased level of GSH was responsible for the MDSCs differentiation.

To gain insight into the mechanisms by which acitretin regulated the expression of GSS, we focused on the transcriptome profiling in the acitretin-treated MDSCs. As we mentioned above, the MAPK signaling pathway was activated in MDSCs treated with acitretin (Figure 4C). To explore whether acitretin induced the differentiation of MDSCs via the MAPK signaling pathway, MDSCs were isolated from the bone marrow of IMQ-induced model mice and were cultured in the presence of GM-CSF with or without acitretin. The results found that acitretin did not affect the p-p38. In contrast, acitretin substantially activated p-MEK1/2, MEK1/2, and p-ERK1/2 (Figure 5E), indicating that the effect of acitretin on GSS expression might through the ERK1/2 MAPK signaling pathway. To address this hypothesis, we treated MDSCs with selumetinib, a specific inhibitor of MEK1/2 (40), which blocks the phosphorylation of ERK1/2 (Figure 5F). We found that inhibition of p-ERK1/2 prevented the expression of GSS in MDSCs (Figure 5F). To evaluate the role of p-ERK1/2 in acitretin-promoted MDSCs differentiation, MDSCs were cultured for 5 days in the presence of GM-CSF and acitretin with or without selumetinib. Consistent with the previous observation, inhibition of p-ERK1/2 abrogated the effect of acitretin on the differentiation of MDSCs into F4/80^+^ macrophages, CD206^+^ M2 macrophages, and CD11c^+^MHC II^+^ dendritic cells, although had no effect on the differentiation of MDSCs into CD86^+^ M1 macrophages (Figure 5G). Collectively, these data indicated that acitretin increased the expression of GSS via the ERK1/2 MAPK signaling pathway.

## Discussion

The significant finding of this study is that acitretin promoted the differentiation of MDSCs in the treatment of psoriasis. Prior to our study, the consensus view on the effect of acitretin on psoriasis was that it inhibited the proliferation of keratinocytes and regulated its differentiation (1). However, our findings suggested that the critical role of acitretin on MDSCs in the treatment of psoriasis. Acitretin decreased the number of MDSCs by promoting them to differentiate into macrophages and dendritic cells. Mechanically, acitretin promoted MDSCs differentiation via increasing the GSH production in MDSCs and neutralizing the high level of ROS, which were mediated by the ERK1/2 MAPK signaling pathway. Therefore, in addition to acting as a mediator of keratinocytes, our study suggests that acitretin plays a crucial role in the differentiation of MDSCs in the treatment of psoriasis.

This study identified that the number of MDSCs and M-MDSCs increased in the peripheral blood and skin lesions of psoriasis patients compared with healthy control subjects, which was similar to the previous findings (23, 25, 26). However, Soler et al. found there was no statistically significant relationship between the disease severity and the number of MDSCs (26). In our study, we enlarged the enrolled psoriasis patients and further divided the MDSCs into two groups according to the latest surface markers (41). We observed that the expansion of MDSCs, especially M-MDSCs (HLA-DR^−^CD11b^+^CD33^+^CD15^−^CD14^+^), in the PBMCs positively correlated with disease severity, while there was no significant correlation between the number of G-MDSCs (HLA-DR^−^CD11b^+^CD33^+^CD15^+^CD14^−^) and disease severity. Therefore, our study further confirmed the critical role MDSCs played in the pathogenesis of psoriasis, especially M-MDSCs.

There is overwhelming evidence that ATRA decreased the number of MDSCs in cancer patients and could differentiate MDSCs into mature myeloid cells (42–44). However, the role of acitretin on MDSCs in psoriasis is still unknown. In this study, our findings showed acitretin reduced the number of MDSCs and M-MDSCs in psoriasis patients and psoriasis-like model mice. In addition, acitretin promoted MDSCs to differentiate into macrophages, especially CD206^+^ M2 macrophages, and CD11c^+^MHC-II^+^ dendritic cells, while inhibited MDSCs differentiate into CD86^+^ M1 macrophages *in vitro*. Acitretin-treated the skin lesions of IMQ-induced model mice further confirmed that the expression of CD206 and MHC-II increased in the skin lesion after the treatment of acitretin, while the expression of CD86 significantly decreased. Macrophages are highly plastic, exhibiting different phenotypes ranging from pro-inflammatory M1 to anti-inflammatory M2 phenotype (45, 46). Besides, conventional DC2s, preferentially express MHC-II, were required for Th2 rather than Th1 cells differentiation (47). Therefore, the differentiation of MDSCs induced by acitretin might further regulate the imbalance of immune cells in skin lesions of psoriasis, which might synergistically inhibit inflammation.

In this study, GSEA analysis of transcriptional profiling of acitretin-treated MDSCs found signaling pathways were enriched in glutathione metabolism. Moreover, acitretin induced the expression of GSS, increased GSH production, and neutralized the ROS level in MDSCs. Interrupting GSH synthesis abolished the effect of acitretin on MDSCs differentiation. Previous researches reported that ROS was essential to maintain the undifferentiated state of MDSCs (38). H_2_O_2_ scavenging induced immature myeloid cells to differentiate into macrophages in tumor-bearing mice (48). GSH, the most important antioxidant in cells, are responsible for the differentiation of MDSCs isolated from tumor-bearing mice by neutralization of ROS (27, 49, 50). Therefore, this previous evidence supported our finding that the increased level of GSH in MDSCs induced by acitretin was responsible for the MDSCs differentiation. However, the precise molecular mechanism of GSH on MDSCs differentiation remains to be elucidated.

Retinoic acid mediated the specific effects of cells by regulating the MAPK signaling pathway. It has been reported that ATRA inhibited proliferation and migration, and repressed p53-dependent apoptosis through inhibition of the MAPK signaling pathway, including p38 MAPK, JNK1/2, and ERK1/2 (51–53). However, ATRA promoted the differentiation of immature cells via activating the ERK1/2 MAPK signaling pathway. For instance, MEK/ERK signaling pathway was activated and regulated in ATRA-induced differentiation of acute promyelocytic leukemia (54). ERK1/2 MAPK signaling pathway, but not of the JNK, p38 MAPK, was essential for the ATRA effects on MDSCs differentiation (55), which was similar to our findings. In this study, transcriptional profiling of acitretin-treated MDSCs found differentially expressed genes enriched in the MAPK signaling pathways. Besides, we found acitretin dramatically increased phosphorylation of MEK1/2 and ERK1/2 in MDSCs but had no effect on the phosphorylation of p38. Inhibition of p-ERK1/2 completely abrogated the effect of acitretin on GSS expression and MDSCs differentiation. These data indicated that acitretin might regulate GSS expression and MDSCs differentiation via ERK activation.

In summary, the present study provided evidence demonstrating that an increased number of MDSCs was found in psoriasis, and acitretin reduced the number of MDSCs in the treatment of psoriasis. Furthermore, acitretin promoted the differentiation of MDSCs via activating the ERK1/2 MAPK signaling pathway, which contributed to the increased expression of GSS and accumulation of GSH in these cells. GSH neutralized the level of ROS in MDSCs and was responsible for acitretin-induced MDSCs differentiation. These results indicated the novel biological mechanisms underlying the effects of acitretin on psoriasis.

## RNA Sequencing Data

The accession number for the sequencing data in this paper is PRJNA711342.

## Data Availability Statement

The datasets generated for this study can be found in online repositories. The names of the repository/repositories and accession number(s) can be found in the article/Supplementary Material.

## Ethics Statement

The studies involving human participants were reviewed and approved by Xiangya hospital of Central South University. Written informed consent to participate in this study was provided by the participants' legal guardian/next of kin. The animal study was reviewed and approved by Xiangya medicine school of Central South University.

## Author Contributions

PL performed all experiments and data analysis and wrote the manuscript. CP and XC contributed to study design. LW assisted in mouse experiments. MY assisted in flow data analysis. JL performed histological analysis. QQ involved in patient recruitment and sample collection. YK and WZ designed and supervised all experiments and wrote the paper. All authors contributed to the article and approved the submitted version.

## Conflict of Interest

The authors declare that the research was conducted in the absence of any commercial or financial relationships that could be construed as a potential conflict of interest.

## Abbreviations

MDSCs: Myeloid-derived suppressor cells
M-MDSCs: Monocytic myeloid-derived suppressor cells
G-MDSCs: Granulocytic myeloid-derived suppressor cells
IMQ: Imiquimod
RA: Retinoid analogs
ATRA: all-trans retinoic acid
PBMCs: peripheral blood mononuclear cells
PASI: psoriasis severity index score
DCs: dendritic cells
PCNA: proliferating cell nuclear antigen
K17: cytokeratin 17
K10: cytokeratin 10
GSS: glutathione synthase
GSH: glutathione
ROS: reactive oxygen species
ERK1/2: extracellular signal-regulated kinase 1/2
MAPK: mitogen-activated protein kinase
IOD: integrated optical density.
