# Supplementary material for: Acitretin Promotes the Differentiation of Myeloid-Derived Suppressor Cells in the Treatment of Psoriasis
Source: Front Med (Lausanne). 2021 Mar 23;8:625130. doi: 10.3389/fmed.2021.625130 (PMC8021725; doi:10.3389/fmed.2021.625130)
Supplement: Supplementary file 1 [file Data_Sheet_1.pdf]

Supplementary Material

1 Supplementary Figures and Tables

1.1 Supplementary Figures

A

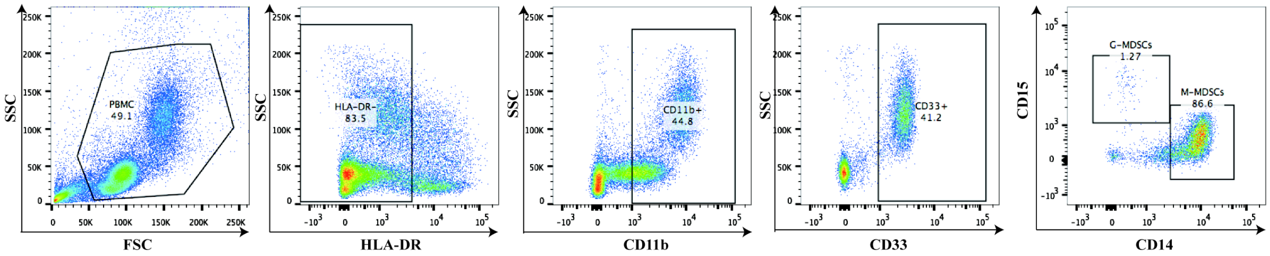

Supplementary Figure 1. Staining profiles of MDSCs in psoriasis patients

(A) Staining profiles of MDSCs (HLA-DR$^-$CD11b$^+$CD33$^+$), M-MDSCs (HLA-DR$^-$CD11b$^+$CD33$^+$CD15$^-$CD14$^+$), and G-MDSCs (HLA-DR$^-$CD11b$^+$CD33$^+$CD15$^+$CD14$^-$) from a representative psoriasis patient.

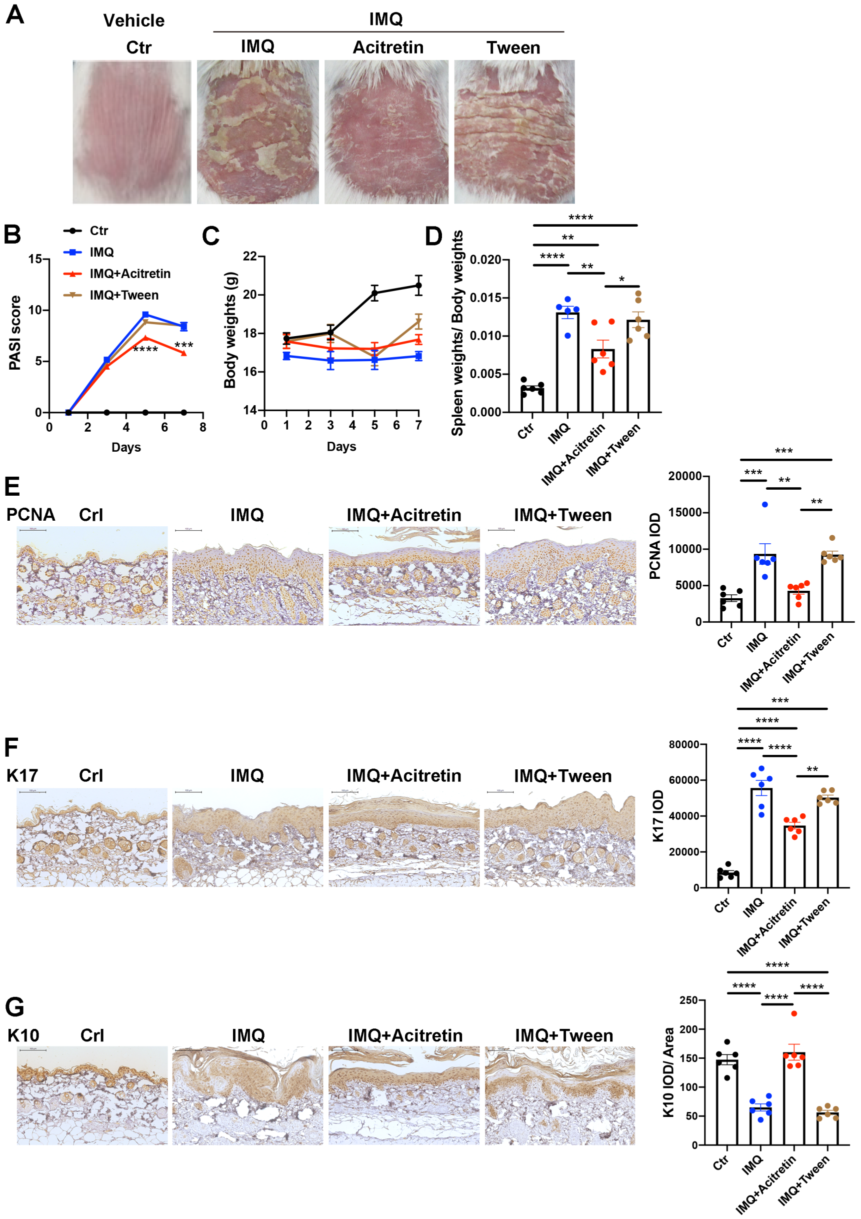

Supplementary Figure 2. Acitretin improved the skin phenotype of the IMQ-induced psoriasis-like model mice

IMQ-induced psoriasis-like model mice treated with oral acitretin or tween (solvent) once per day for 6 days. **(A)** The clinical manifestations of the back skin derived from Control (Ctr) and IMQ-induced model mice treated with acitretin or tween (solvent) (n=6). PASI score **(B)**, body weights **(C)**, and spleen weights/ body weights **(D)** of mice in **A** (n=6). Paraffin sections of the back skin of Control (Ctr), IMQ, IMQ+Acitretin, and IMQ+Tween group were stained for PCNA, K17, and K10 by immunohistochemistry. PCNA IOD, K17 IOD, and K10 IOD/ Area measured by image pro plus 6.0 expressed the PCNA, K17, and K10 expression. **(E)** PCNA stain, **(F)** K17 stain, **(G)** K10 stain. Scale bars: 100 μm. Statistical data are shown in the right panel. All results represent at least 3 independent experiments. Data represent the mean ± SEM. $*p < 0.05$, $**p < 0.01$, $***p < 0.001$, $****p < 0.0001$.

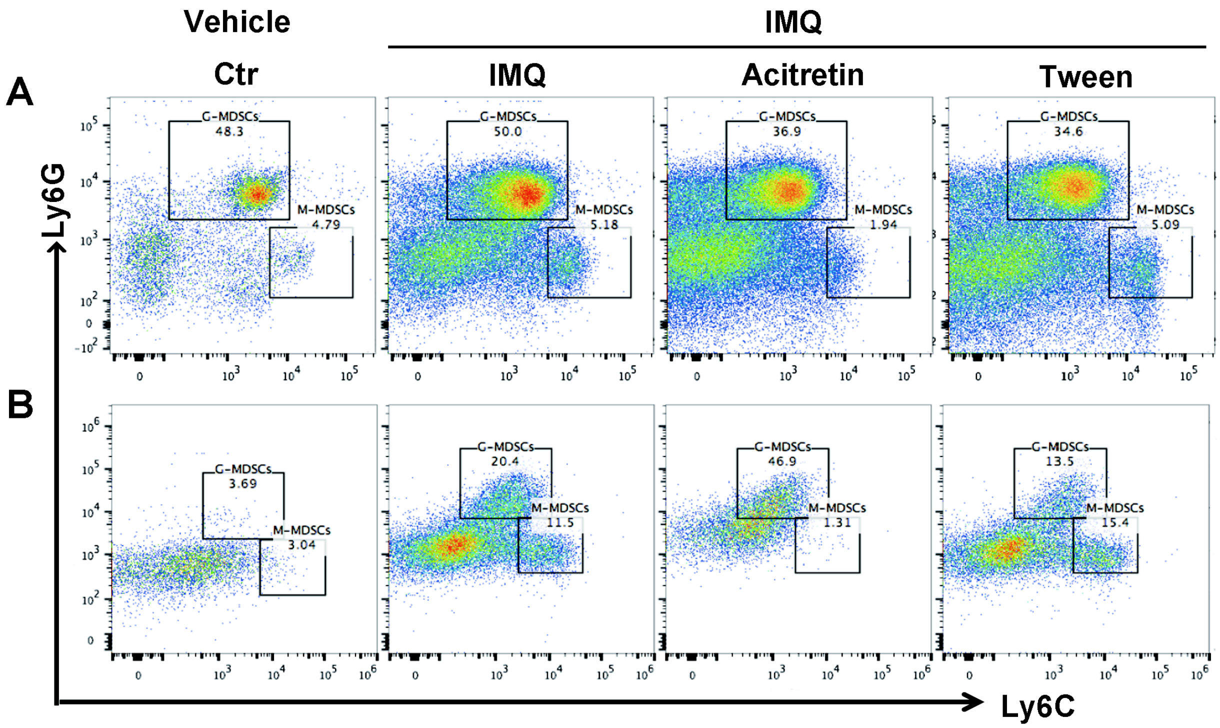

Supplementary Figure 3. Staining profiles of MDSCs, M-MDSCs, and G-MDSCs in the IMQ-induced psoriasis-like model mice

Representative flow cytometric analysis of Gr-1^{+}CD11b^{+} MDSCs, CD11b^{+}Ly6G^{-}Ly6C^{+} M-MDSCs, and CD11b^{+}Ly6G^{+}Ly6C^{-} G-MDSCs in the spleen **(A)** and skin lesion **(B)** of IMQ-induced psoriasis-like model mice treated with oral acitretin or tween (solvent).

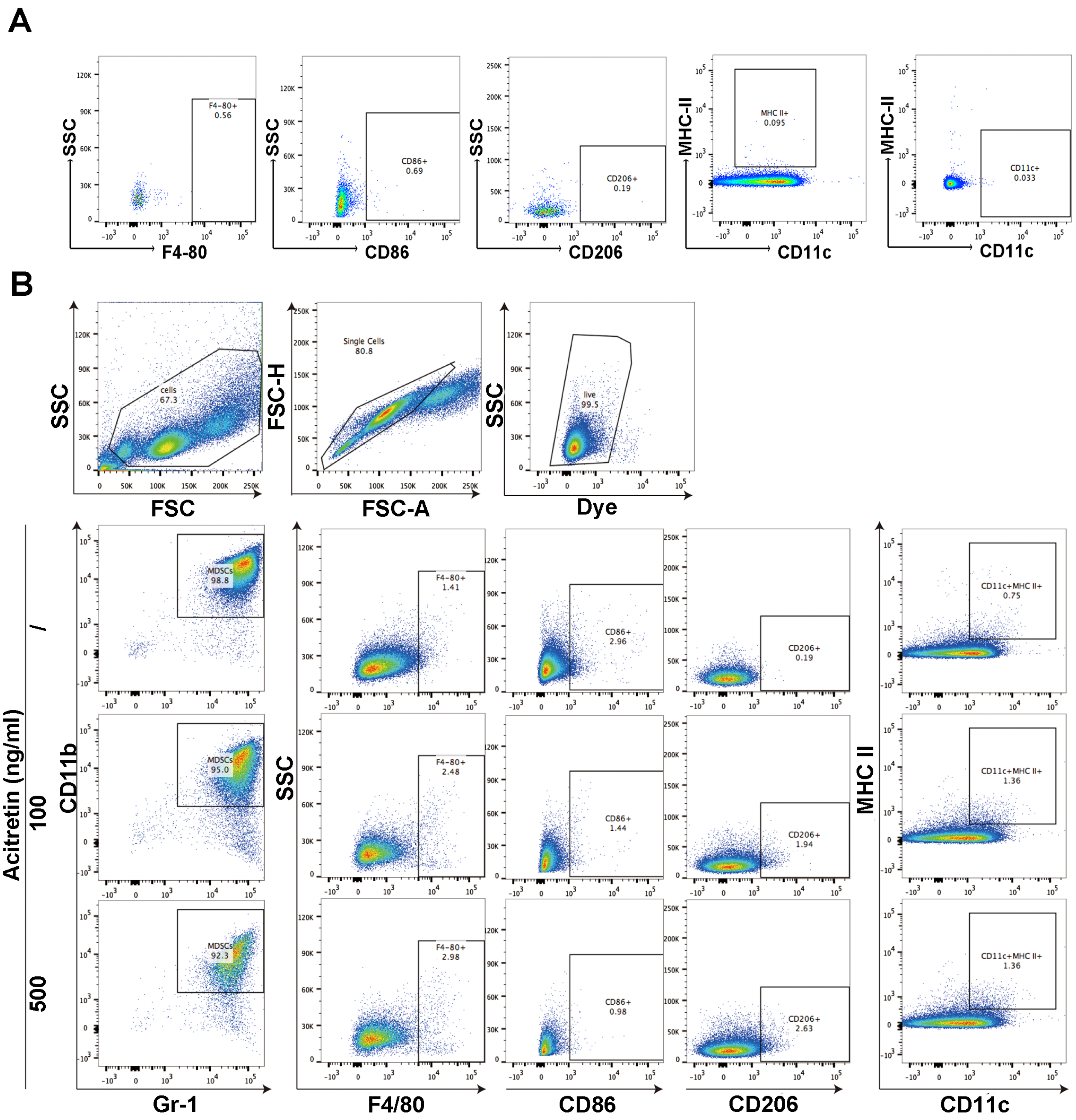

Supplementary Figure 4. Acitretin promoted the differentiation of MDSCs

(A) Fluorescence minus one (FMO) control was used to set gates for F4/80, CD86, CD206, MHC-II, and CD11c in differentiation experiments. **(B)** Representative flow cytometric analysis of Gr-1^+CD11b$^+$ MDSCs, F4/80$^+$ macrophage, CD86$^+$ M1 macrophage, CD206$^+$ M2 macrophage, and CD11c$^+$MHCII$^+$ dendritic cells.

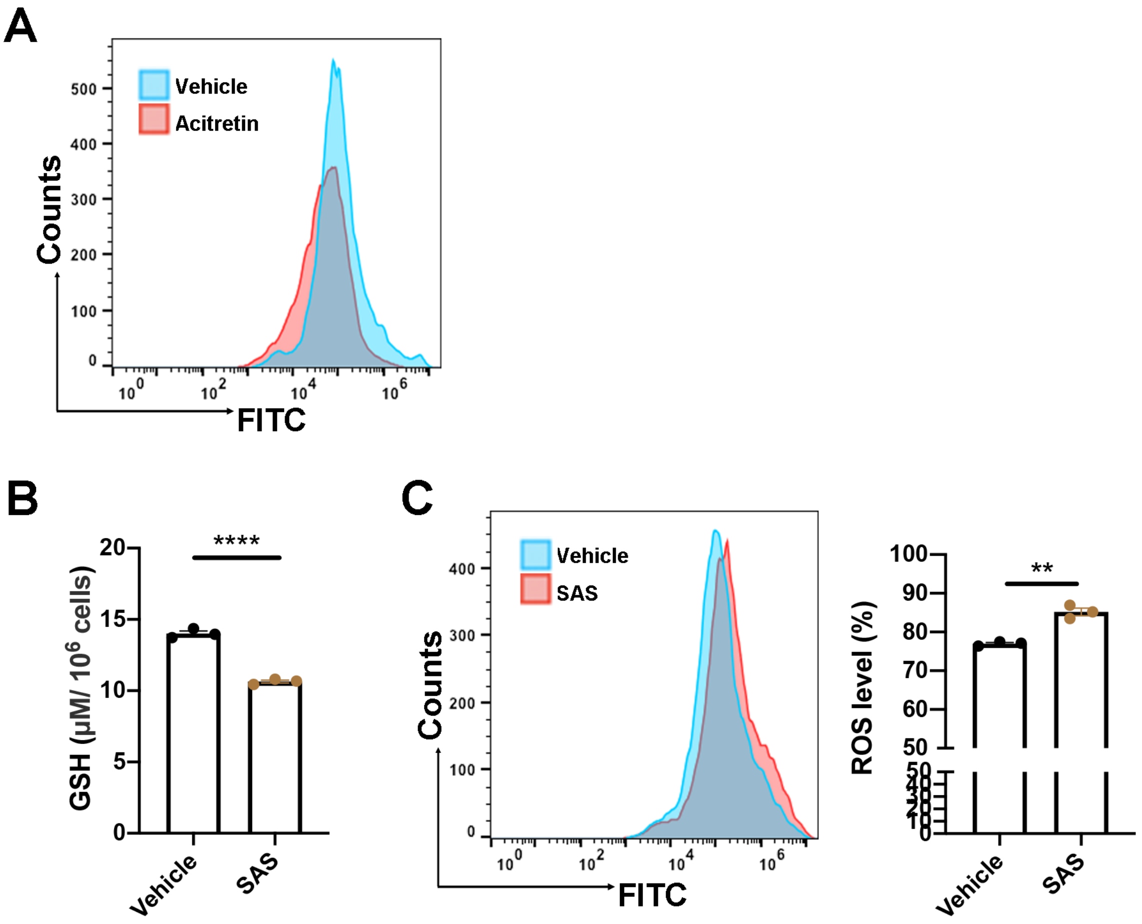

Supplementary Figure 5. Mechanism of acitretin effect on the differentiation of MDSCs

(A) Representative ROS flow cytometric analysis. Gr-1$^+$MDSCs were isolated from the bone marrow of IMQ-induced model mice and cultured in the presence of 20 ng/ml GM-CSF with vehicle control or 500 ng/ml acitretin for 48h. MDSCs were obtained, and the level of ROS was detected by flow cytometry. **(B-C)** Gr-1$^+$MDSCs were isolated from the bone marrow of IMQ-induced model mice and cultured in the presence of 20 ng/ml GM-CSF with vehicle control or 200 μM SAS for 48h. MDSCs were obtained, and the level of GSH was measured with a GSH detection kit. The level of ROS was detected by flow cytometry. **(B)** The level of GSH in MDSCs. **(C)** Representative ROS flow cytometric analysis of MDSCs (left panel). The level of ROS in MDSCs (right panel). Data represent the mean ± SEM. $^{**}p < 0.01$, $^{****}p < 0.0001$.

Supplementary Table 1. Demographic information of the subjects who donated peripheral blood

	Health controls (Ctr)	Plaque psoriasis (Pso)
Number	30	77
Age (y)	40.50 ± 1.918	41.42 ± 1.492
Sex (F/M)	11/19	19/58
BMI (kg/m^2)	-	24.32 ± 0.4789
Age of the first onset (y)	-	35.61 ± 1.636
Duration (m)	-	68.26 ± 7.754
Comorbidities	-	10 (13.0%)
Family history	-	7 (9.1%)
Somking history	-	22 (28.6%)
PASI score	-	9.705 ± 0.6801

Values are Mean ± SEM. BMI: body mass index.

Supplementary Table 2. Demographic information of the subjects who donated skin

	Health controls (Ctr)	Plaque psoriasis (Pso)
Number	9	20
Age (y)	44.67 ± 3.037	37.68 ± 3.213
Sex (F/M)	5/4	6/14
BMI (kg/m^2)	-	23.04 ± 0.9538
Age of the first onset (y)	-	32.23 ± 3.386
Duration (m)	-	44.85 ± 18.08
Comorbidities	-	2 (10.0%)
Family history	-	1 (5.0%)
Somking history	-	6 (30.0%)
PASI score	-	9.420 ± 2.025

Values are Mean ± SEM. BMI: body mass index.

Supplementary Table 3. Demographic information of the psoriasis patients treated with acitretin for 8 weeks

	Plaque psoriasis (Pso)
Number	17
Age (y)	41.18 ± 3.023
Sex (F/M)	4/13
BMI (kg/m^2)	23.12 ± 0.8127
Age of the first onset (y)	34.38 ± 2.234
Duration (m)	78.50 ± 20.73
Comorbidities	3 (17.6%)
Family history	0 (0.0%)
Somking history	4 (23.5%)
PASI score before the treatment	13.99 ± 1.867
PASI score after the treatment	7.817 ± 1.179 [A]

Values are Mean ± SEM. BMI: body mass index.

[A] $p < 0.05$.
